# Biopsy-Proven Gastric Pathological Findings in Mechanically Ventilated Intensive Care Unit Patients

**DOI:** 10.7759/cureus.61744

**Published:** 2024-06-05

**Authors:** Elvan Tekir Yılmaz, Bilge Olgun Keleş

**Affiliations:** 1 Anesthesiology and Reanimation, Giresun University the Faculty of Medicine, Giresun, TUR

**Keywords:** mechanical ventilation, intensive care units, peptic ulcer, helicobacter pylori, gastrointestinal hemorrhage

## Abstract

Objectives

*Helicobacter pylori (H. pylori*) is known to affect a large proportion of the world population. It plays a role in the pathogenesis of peptic ulcer (PU) and increases the likelihood of bleeding. In critically ill patients in intensive care units (ICUs), the risk of bleeding may be much higher due to many concomitant factors. The study aimed to determine the activation of *H. pylori* in mechanically ventilated (MV) intensive care patients and compare this with the general population.

Methods

This study was performed retrospectively by screening patients who underwent esophagogastroduodenoscopy and histopathological sampling in our hospital between January and June 2023. The study included 79 patients aged between 18 and 85 years. The patients were categorized into two groups: 35 patients in the ICU with mechanical ventilation (MV) support (E_MV_) and 44 patients who presented to the gastroenterology department due to dyspeptic symptoms and underwent endoscopy (_ED_). Age; sex characteristics; laboratory parameters such as hemoglobin (Hb), hematocrit (Htc), mean cellular volume (MCV), white blood cell (WBC), neutrophil, platelet, glucose, urea, creatinine, aspartate transaminase (AST), alanine transaminase (ALT), C-reactive protein (CRP), albumin, ferritin, thyroid-stimulating hormone (TSH), anti-hepatitis C virus (HCV), hepatitis B surface antigen (HBsAg), anti-HIV; and biopsy results (*H. pylori* positivity, intestinal metaplasia, and atrophy) were recorded.

Results

A total of 79 patients who underwent gastric biopsy were assessed. There were 35 patients in the E_MV_ group and 44 patients in the E_D _group. There was no difference in gender and age distribution between the groups. Hb and Htc were significantly lower in E_MV_ compared to E_D_ (p=0.001). Hb was 9.4±1.7 g/dL in E_MV_ and 10.8±2.1 g/dL in E_D_. Htc was 29.6±5.1 in E_MV_ and 33.5±5.7 in E_D_. MCV, WBC, glucose, urea, AST, ALT, CRP, and ferritin values were statistically significantly higher in E_MV_ (p<0.05). Albumin and creatinine levels were statistically significantly lower in E_MV_ (p<0.05). There was no significant difference between the groups in terms of neutrophils, platelets, and TSH. In the E_MV_ group, *H. pylori* activity was negative in 31 (88.6%) patients and positive in four (11.4%) patients. In the E_D _group, *H. pylori* activity was negative in 30 (68.2%) patients and positive in 14 (31.8%) patients. There was a statistically significant difference between the groups in terms of *H. pylori* positivity (p=0.032).

Conclusions

The prevalence of *H. pylori* in MV patients in the ICU is low compared to the average population. The incidence of atrophic gastritis and intestinal metaplasia is the same. The present study supports that ICU cases do not have a higher risk of gastric premalignant lesions compared to the average population.

## Introduction

Peptic ulcer disease (PUD) represents a significant concern for the general population, as well as for patients in the intensive care unit (ICU). This concern may be slightly higher in ICU patients for several reasons. The presence of pre-existing comorbidities also increases the risk of developing PUD [1]. Comorbidity is included in scoring systems predicting mortality from upper gastrointestinal (GI) bleeding and increases risk [2,3]. One of the factors leading to augmented risk in the ICU is age. The risk of upper GI bleeding in cases over 60 years is 10 times higher than in younger ones. As such, mortality is also higher in this age group [4]. The risk of peptic ulcer (PU) bleeding in these patients is also increased by the use of multiple medications, antiplatelet, anticoagulant, and corticosteroid treatment. Gastric mucosal ischemia is also an essential factor leading to mucosal damage, which factors leads to stress ulcers and bleeding in 1-9% of ICU cases [5]. *Helicobacter pylori* (*H. pylori*) is the most common cause of PU, and studies have shown that it doubles the risk of bleeding from it [6-8]. *Helicobacter pylori* infection contributes to the development of PU by disrupting the mucus structure and mucosal integrity of the stomach [9]. Eradication of *H. pylori* has been shown to reduce the development of PU [10,11]. The use of proton pump inhibitors (PPIs) is quite common in the ICU, with no effect on length of stay, pneumonia risk, and mortality, while reducing the risk of GI bleeding by 50% [12]. There are studies suggesting that all patients in the ICU with or without MV (mechanical ventilation) should receive gastroprotective therapy [13]. Although multiple factors contribute to the development of GI bleeding in critically ill patients by disrupting mucosal integrity, the incidence is reported to be low compared to the average population [14]. As such, gastroprotection and low prevalence of *H. pylori *in ICU cases might make these outcomes likely. Nevertheless, no study based on gastric biopsy results has been reported to date. The present study aimed to demonstrate *H. pylori *activity in histopathological samples obtained by endoscopy in cases followed up in the ICU with MV. We believe that the results compared with samples from the normal population will shed light on this discussion.

## Materials and methods

This study was approved by the local Training and Research Hospital Clinical Research Ethics Committee. In this study, the results of patients who underwent gastric biopsy for reasons such as percutaneous endoscopic gastrostomy opening, gastric intolerance and bleeding while being followed up in the ICU with MV support of a tertiary education and research hospital between January and June 2023 were retrospectively compared with the results of daily patients who underwent gastric biopsy for dyspeptic complaints in the endoscopy unit. Thirty-five ICU patients on MV were defined as E_MV_, and 44 patients who presented with dyspeptic complaints and underwent upper GI endoscopy were defined as E_D_. Exclusion criteria were as follows: i) under 18 and over 85 years of age, ii) patients with bleeding with impaired hemodynamic parameters, iii) patients with more than four units of erythrocyte suspension replacement, iv) patients with a diagnosis of gastric cancer, and v) patients with previous gastric surgery. Age, sex characteristics, laboratory parameters such as hemoglobin (Hb), hematocrit (Htc), mean cell volume (MCV), white blood cell (WBC), neutrophil, platelet, glucose, urea, creatinine, aspartate transaminase (AST), alanine transaminase (ALT), C-reactive protein (CRP), albumin, ferritin, thyroid stimulating hormone (TSH), anti-hepatitis C virus (HCV), hepatitis B surface antigen (HBsAg), anti-HIV; and gastric biopsy results (*H. pylori* positivity, intestinal metaplasia, and atrophy) were recorded. Furthermore, data pertaining to the antibiotics administered to ICU patients during their hospitalization, the reasons for their hospitalization, and the length of their hospital stay were also recorded. The E_D_ group was formed by selecting patients with complete data among patients who used PPIs and underwent endoscopy for dyspeptic complaints in the same six-month interval. Furthermore, we used PPIs in patients undergoing intensive care with mechanical ventilation (MV) support. In the E_MV_ and E_D_ groups, all patients were on PPIs.

Statistical analyses were performed using SPSS Version 23 (IBM Corp., Armonk, NY). Normality analysis of quantitative data was performed using the Kolmogorov-Smirnov test. The comparison of normally distributed data was performed using the independent sample t-test, and the comparison of non-normally distributed data was performed using the Mann-Whitney U test. Qualitative data were compared using the Pearson chi-square test. Data were presented as n (%) and mean (±) standard deviation. The relationships between the data were analyzed by the Pearson correlation analysis. The statistical significance value was accepted as p<0.05.

## Results

The mean age was 71±17 years in E_MV_ and 75.1±9.5 in E_D_, which was not significantly different (p=0.577) (Table 1). There was no statistically significant difference between the two groups, although there were more men in E_MV_ and more women in E_D_ (Table 2). The Hb value was 9.4±1.7 g/dL in E_MV_ and 10.8±2.1 g/dL in E_D_. However, Htc was 29.6±5.1% in E_MV_ and 33.5±5.7% in E_D_. Hb and Htc were significantly higher in E_D_ (p=0.001). In addition, albumin (29.1±5.5 g/dL) and creatinine (0.81±0.67 mg/dL) in E_MV _were significantly lower than in E_D _(p<0.00 and p=0.014, respectively), whereas MCV, WBC, glucose, urea, AST, ALT, CRP, and ferritin levels were significantly higher in E_MV_ (p<0.05). Neutrophil, platelet, and TSH levels were not different between them (Table 1).

**Table 1:** Patients' laboratory results MCV, mean corpuscular volume; WBC, white blood cell; AST, aspartate aminotransferase; ALT, alanine aminotransferase; CRP, C-reactive protein; TSH, thyroid-stimulating hormone

	E_MV_		E_D_		p-Value
	n	Mean ± SD	n	Mean ± SD	
Age	35	71 ± 17	44	75.1 ± 9.5	0.577
Hemoglobin (g/dL)	35	9.4 ± 1.7	44	10.8 ± 2.1	0.001
Hematocrit (%)	35	29.6 ± 5.1	44	33.5 ± 5.7	0.001
MCV (fL)	35	93.6 ± 7.2	44	85.4 ± 6.6	<0.001
WBC (/mcL)	35	9.79 ± 4.55	44	7.65 ± 2.44	0.020
Neutrophil (/mcL)	35	70 ± 20.9	44	62.7 ± 24.9	0.064
Platelet (/mm^3^)	35	268 ± 120	44	256 ± 97	0.393
Glucose (mg/dL)	35	129 ± 46	44	104 ± 24	0.006
Urea (mg/dL)	35	73 ± 40	44	47 ± 35	0.001
Creatinine (mg/dL)	35	0.81 ± 0.67	44	0.97 ± 0.64	0.014
AST (U/L)	35	34 ± 29	44	21 ± 8	0.009
ALT (U/L)	35	26 ± 22	44	16 ± 10	0.025
CRP (mg/dL)	35	80.08 ± 76.73	44	25.61 ± 48.76	<0.001
Albumin (g/dL)	35	29.1 ± 5.2	44	38.7 ± 7.7	<0.001
Ferritin (mL/ng)	35	690.5 ± 534.5	44	114.3 ± 187.7	<0.001
TSH (mIU/L)	35	2.29 ± 4.59	44	1.57 ± 1.32	0.945

In E_MV_, 31 (88.6%) cases were negative and four (11.4%) were positive in *H. pylori* activity, while in E_D_, 30 (68.2%) patients were negative and 14 (31.8%) were positive in *H. pylori *activity. A significant difference in *H. pylori* positivity (p=0.032) and no difference in the frequency of intestinal metaplasia and atrophic gastritis were recognized (p=0.220, p=0.666, respectively). The number of cases with chronic inflammation was higher in the E_MV_ group (p<0.001). However, the anti-HCV and anti-HIV positivity did not differ (p=0.201, p=0.108, respectively), while HBsAg positivity was higher in E_D_ (p=0.039) (Table 2). In the correlation analysis, a low-level negative correlation was detected between MCV and *H. pylori* positivity (Table 3). Antibiotics administered to ICU patients during hospitalization, reasons for hospitalization, and length of hospital stay are shown in Table 4.

**Table 2:** Gastric biopsy and ELISA results HCV, hepatitis C virus; HBsAg, hepatitis B surface antigen; ELISA, enzyme-linked immunosorbent assay

	E_MV,_ n (%)	E_D, _n (%)	p-Value
Sex (female/male)	15 (42.9)/20 (57.1)	25 (56.8)/19 (43.2)	0.218
Anti-HCV (+)	0 (0)	2 (4.5)	0.201
HBsAg (+)	0 (0)	5 (11.4)	0.039
Anti-HIV (+)	2 (5.7)	0 (0)	0.108
*Helicobacter pylori *(+)	4 (11.4)	14 (31.8)	0.032
Intestinal metaplasia (+)	3 (8.6)	8 (18.2)	0.220
Atrophy (+)	6 (17.1)	6 (13.6)	0.666
Chronic inflammation (+)	33 (94.3)	2 (4.5)	<0.001

**Table 3:** Correlation analysis of H. pylori positivity with laboratory data MCV, mean corpuscular volume; WBC, white blood cell; AST, aspartate aminotransferase; ALT, alanine aminotransferase; CRP, C-reactive protein; TSH, thyroid-stimulating hormone

	E_MV_		E_D_	
	r	p-Value	r	p-Value
Age	0.015	0.930	-0.202	0.189
Hemoglobin (g/dL)	0.039	0.824	0,057	0.712
Hematocrit (%)	0.097	0.581	0,079	0.610
MCV (fL)	-0.360	0.034	-0.087	0.574
WBC (/mcL)	-0.115	0.509	0.019	0.905
Neutrophil (/mcL)	0.041	0.815	-0.159	0.303
Platelet (/mm^3^)	0.076	0.663	-0.254	0.096
Glucose (mg/dL)	-0.132	0.450	0.024	0.877
Ürea (mg/dL)	0.126	0.470	-0.294	0.053
Creatinine (mg/dL)	0.406	0.016	-0.203	0.187
AST (U/L)	-0.128	0.463	0.223	0.145
ALT (U/L)	0.062	0.724	0.200	0.194
CRP (mg/dL)	-0.043	0.806	0.030	0.847
Albumin (g/dL)	-0.010	0.954	0.032	0.839
Ferritin (mL/ng)	-0.129	0.461	0.230	0.134
TSH (mIU/L)	-0.028	0.875	0.214	0.164

**Table 4:** Characteristics of patients in the ICU ICU, intensive care unit; RS, respiratory system; CNS, central nervous system; CVS, cardiovascular system; CPR, cardiopulmonary resuscitation

	E_MV _(n=35)
Length of ICU stay (mean±SD)	39.29±16.02
Reason for ICU hospitalization (n)	
RS diseases	4
CNS diseases	20
CVS diseases	2
Post-CPR	9
Used antibiotics (n)	
Penicillin group antibiotics	12
Tetracycline group antibiotics	4
Quinolone group antibiotics	15
Other antibiotics	15

## Discussion

The present study retrospectively evaluated 79 cases who had undergone gastric biopsy and found that the prevalence of *H. pylori *was lower in ICU patients than in the average population with dyspeptic complaints. The incidence of atrophic gastritis and intestinal metaplasia did not differ between the groups.

Gastric biopsy remains the most valid option to determine the frequency of *H. pylori* infection. However, the usability of each method on different patient populations is limited. The selection of the method is influenced by the patient's general condition, location within the hospital, and the invasiveness of the procedure. The diagnosis of *H. pylori* infection is based on non-invasive methods such as serology, 13C-urea breath test, stool antigen test, and methods requiring gastric biopsy during endoscopy (histopathology, culture, rapid urease test, polymerase chain reaction) [15]. The rapid urease test is specific but less sensitive (80-90%). The 13C-urea breath test is difficult to perform in ICU patients, especially those on MV. The performance of the fecal antigen test is close to that of the urea breath test and might be more suitable for use in ICU cases [16]. Serological tests are inexpensive and are mostly used for screening. However, there is insufficient evidence to support the use of any of these tests in place of histopathological diagnosis [17,18]. Biopsy culture helps to determine antimicrobial resistance and susceptibility and to guide treatment planning. It allows the identification of mucosal lesions and gastric inflammatory processes [15]. Therefore, endoscopy seems to be the most valid and effective method.

Stress-related gastrointestinal mucosal injury is most prevalent in patients with acute hepatic failure, anticoagulant use, severe burns, coagulopathy, lack of enteral nutrition, recent gastroduodenal ulcer, corticosteroid use, *H. pylori *infection, neurological injury, trauma, nonsteroidal anti-inflammatory drug use, mechanical ventilation, shock, and sepsis. Furthermore, it has also been found that the presence of *H. pylori* immunoglobulin antigen detected by the ELISA (enzyme-linked immunosorbent assay) method was a contributing factor for upper GI bleeding in cases treated in the ICU [17]. However, the prevalence of gastric premalignant disorders such as atrophic gastritis, intestinal metaplasia, and *H. pylori *infection remains controversial, especially among patients with MV.

Robert et al. conducted a prospective, multicenter, epidemiological study that enrolled 1,776 ICU patients and reported that *H. pylori* antigen seropositivity detected by rectal swab (fecal antigen test) was higher in female subjects than their male counterparts [16]. In our study, we found no difference between the two groups in terms of gender and age.

In a Dutch prospective observational study of 300 consecutive MV cases, the prevalence of *H. pylori* infection decreased from 38% during hospitalization to 0% one week later, probably due to the use of intensive antibiotic therapy for intestinal decontamination [11]. In contrast, a study of 100 ICU patients found that the rate of *H. pylori *infection was higher than in controls, regardless of age and disease severity scale or stress ulcer risk score [18]. Furthermore, a large-scale ICU study including 4,341 patients reported that *H. pylori *infection was more frequent in patients with bleeding than in matched controls [19]. In this study, we found a low prevalence of *H. pylori *in patients with MV and hypothesized that the use of antibiotics to treat other diseases may have contributed to this finding. However, there has not been a study including both atrophic gastritis and intestinal metaplasia among ICU patients with MV. In this study, no difference was found between the E_MV_ group and the E_D_ group. Based on these findings, we concluded that patients followed with MV do not constitute an additional risk factor for gastric premalignant diseases. Nguyen et al. reported that hemoglobin concentration decreased >0.5 g/dL/day during the first days of ICU stay, and this decrease continued in cases with high APACHE II scores [20]. In our study, Hb and Htc values were lower in E_MV_. This may contribute to perfusion impairment and ischemia by decreasing oxygen supply to tissues. In this study, high WBC, CRP, and ferritin levels in patients on MV can be attributed to the existing infections of the patients. AST and ALT elevations may be associated with the drugs used and hypoxia.

In the present study, GI bleeding occurred in only two patients in the E_MV_ group. Similar to other studies (1-9%), the incidence of bleeding was 5.8% [5]. The decreased incidence of bleeding in the ICU compared to the average population suggests that it is a factor that may reverse the disadvantage caused by factors such as mucosal ischemia, age, and anticoagulant use. In addition to the widespread use of PPIs as stress ulcer prophylaxis, it may be due to the low prevalence of *H. pylori*.

Limitations

This study retrospectively analyzed patients on mechanical ventilation in the ICU who underwent endoscopic intervention. As this procedure was performed in conformity with the indication, the number of cases is limited.

## Conclusions

The prevalence of *H. pylori *infection detected by gastric biopsy was low in ICU cases. Of note, the presence of atrophic gastritis and intestinal metaplasia was similar in both E_D_ and E_MV_. Therefore, the results of our preliminary report support that despite many stressors, cases in the ICU on MV do not have a higher risk of gastric premalignant lesions compared to the average population.
